# Supplementary material for: Coupled symmetric and asymmetric circuits underlying spatial orientation in fruit flies
Source: Nat Commun. 2017 Jul 26;8:139. doi: 10.1038/s41467-017-00191-6 (PMC5529380; doi:10.1038/s41467-017-00191-6)
Supplement: Supplementary file 1 — Supplementary Information [file 41467_2017_191_MOESM1_ESM.pdf]

File Name: Supplementary Information
Descriptions: Supplementary Tables

File Name: Peer Review File
Descriptions:

Supplementary Table 1. The innervation table of EIP, PEI and PEN neuron classes

**Neuron class**	**Neuron type**	**PB -R8**	**PB -R7**	**PB -R6**	**PB -R5**	**PB -R4**	**PB -R3**	**PB -R2**	**PB -R1**	**PB -R0**	**PB -L0**	**PB -L1**	**PB -L2**	**PB -L3**	**PB -L4**	**PB -L5**	**PB -L6**	**PB -L7**	**PB -L8**
EIP	EIP0	2																	
EIP	EIP1		2																
EIP	EIP2			2															
EIP	EIP3				2														
EIP	EIP4					2													
EIP	EIP5						2												
EIP	EIP6							2											
EIP	EIP7								2										
EIP	EIP8									2									
EIP	EIP9										2								
EIP	EIP10											2							
EIP	EIP11												2						
EIP	EIP12													2					
EIP	EIP13														2				
EIP	EIP14															2			
EIP	EIP15																2		
EIP	EIP16																	2	
EIP	EIP17																		2
PEI	PEI0		1																
PEI	PEI1			1															
PEI	PEI2				1														
PEI	PEI3					1													
PEI	PEI4						1												
PEI	PEI5							1											
PEI	PEI6								1										
PEI	PEI7									1									
PEI	PEI8										1								
PEI	PEI9											1							
PEI	PEI10												1						
PEI	PEI11													1					
PEI	PEI12														1				
PEI	PEI13															1			
PEI	PEI14																1		
PEI	PEI15																	1	
PEN	PEN0	1																	
PEN	PEN1		1																
PEN	PEN2			1															
PEN	PEN3				1														
PEN	PEN4					1													
PEN	PEN5						1												
PEN	PEN6							1											
PEN	PEN7								1										
PEN	PEN8											1							
PEN	PEN9												1						
PEN	PEN10													1					
PEN	PEN11														1				
PEN	PEN12															1			
PEN	PEN13																1		
PEN	PEN14																	1	
PEN	PEN15																		1

Neuron class	Neuron type	EB-R8C	EB-R7C	EB-R6C	EB-R5C	EB-R4C	EB-R3C	EB-R2C	EB-R1C	EB-L1C	EB-L2C	EB-L3C	EB-L4C	EB-L5C	EB-L6C	EB-L7C	EB-L8C
EIP	EIP0	1															
EIP	EIP1	1	1	1													
EIP	EIP2			1	1	1											
EIP	EIP3					1	1	1									
EIP	EIP4							1	1	1							
EIP	EIP5									1	1	1					
EIP	EIP6											1	1	1			
EIP	EIP7													1	1	1	
EIP	EIP8	1														1	1
EIP	EIP9	1	1														1
EIP	EIP10		1	1	1												
EIP	EIP11				1	1	1										
EIP	EIP12						1	1	1								
EIP	EIP13								1	1	1						
EIP	EIP14										1	1	1				
EIP	EIP15												1	1	1		
EIP	EIP16														1	1	1
EIP	EIP17																1
PEI	PEI0		2	2													
PEI	PEI1				2	2											
PEI	PEI2						2	2									
PEI	PEI3								2	2							
PEI	PEI4										2	2					
PEI	PEI5												2	2			
PEI	PEI6														2	2	
PEI	PEI7	2															2
PEI	PEI8	2															2
PEI	PEI9		2	2													
PEI	PEI10				2	2											
PEI	PEI11						2	2									
PEI	PEI12								2	2							
PEI	PEI13										2	2					
PEI	PEI14												2	2			
PEI	PEI15														2	2	
PEN	PEN0																
PEN	PEN1																
PEN	PEN2																
PEN	PEN3																
PEN	PEN4																
PEN	PEN5																
PEN	PEN6																
PEN	PEN7																
PEN	PEN8																
PEN	PEN9																
PEN	PEN10																
PEN	PEN11																
PEN	PEN12																
PEN	PEN13																
PEN	PEN14																
PEN	PEN15																

Neuron class	Neuron type	EB-R8P	EB-R7P	EB-R6P	EB-R5P	EB-R4P	EB-R3P	EB-R2P	EB-R1P	EB-L1P	EB-L2P	EB-L3P	EB-L4P	EB-L5P	EB-L6P	EB-L7P	EB-L8P
EIP	EIP0	1															
EIP	EIP1	1	1	1													
EIP	EIP2			1	1	1											
EIP	EIP3					1	1	1									
EIP	EIP4							1	1	1							
EIP	EIP5									1	1	1					
EIP	EIP6											1	1	1			
EIP	EIP7													1	1	1	
EIP	EIP8	1														1	1
EIP	EIP9	1	1														1
EIP	EIP10		1	1	1												
EIP	EIP11				1	1	1										
EIP	EIP12						1	1	1								
EIP	EIP13								1	1	1						
EIP	EIP14										1	1	1				
EIP	EIP15												1	1	1		
EIP	EIP16														1	1	1
EIP	EIP17																1
PEI	PEI0																
PEI	PEI1																
PEI	PEI2																
PEI	PEI3																
PEI	PEI4																
PEI	PEI5																
PEI	PEI6																
PEI	PEI7																
PEI	PEI8																
PEI	PEI9																
PEI	PEI10																
PEI	PEI11																
PEI	PEI12																
PEI	PEI13																
PEI	PEI14																
PEI	PEI15																
PEN	PEN0		2	2													
PEN	PEN1				2	2											
PEN	PEN2						2	2									
PEN	PEN3								2	2							
PEN	PEN4										2	2					
PEN	PEN5												2	2			
PEN	PEN6														2	2	
PEN	PEN7	2															2
PEN	PEN8	2															2
PEN	PEN9		2	2													
PEN	PEN10				2	2											
PEN	PEN11						2	2									
PEN	PEN12								2	2							
PEN	PEN13										2	2					
PEN	PEN14												2	2			
PEN	PEN15														2	2	

The table is derived from Lin C.-Y. et al. Cell Rep. 3, 1739-1753 (2013) and Wolff, T., Iyer, N. A. & Rubin, G. M. J. Comp. Neurol. 523, 997-1037 (2015).

1: Postsynaptic terminal

2: Presynaptic terminal

Supplementary Table 2. Connection matrix of the full EB-PB circuits

		Destination																	
		0	1	2	3	4	5	6	7	8	9	10	11	12	13	14	15	16	17
Source		EIP 0	EIP 1	EIP 2	EIP 3	EIP 4	EIP 5	EIP 6	EIP 7	EIP 8	EIP 9	EIP 10	EIP 11	EIP 12	EIP 13	EIP 14	EIP 15	EIP 16	EIP 17
0	EIP0																		
1	EIP1																		
2	EIP2																		
3	EIP3																		
4	EIP4																		
5	EIP5																		
6	EIP6																		
7	EIP7																		
8	EIP8																		
9	EIP9																		
10	EIP10																		
11	EIP11																		
12	EIP12																		
13	EIP13																		
14	EIP14																		
15	EIP15																		
16	EIP16																		
17	EIP17																		
18	PEI0		1	1							1	1							
19	PEI1			1	1							1	1						
20	PEI2				1	1							1	1					
21	PEI3					1	1							1	1				
22	PEI4						1	1							1	1			
23	PEI5							1	1							1	1		
24	PEI6								1	1							1	1	
25	PEI7	1	1							1	1							1	1
26	PEI8	1	1							1	1							1	1
27	PEI9		1	1							1	1							
28	PEI10			1	1							1	1						
29	PEI11				1	1							1	1					
30	PEI12					1	1							1	1				
31	PEI13						1	1							1	1			
32	PEI14							1	1							1	1		
33	PEI15								1	1							1	1	
34	PEN0		1	1							1	1							
35	PEN1			1	1							1	1						
36	PEN2				1	1							1	1					
37	PEN3					1	1							1	1				
38	PEN4						1	1							1	1			
39	PEN5							1	1							1	1		
40	PEN6								1	1							1	1	
41	PEN7	1	1							1	1							1	1
42	PEN8	1	1							1	1							1	1
43	PEN9		1	1							1	1							
44	PEN10			1	1							1	1						
45	PEN11				1	1							1	1					
46	PEN12					1	1							1	1				
47	PEN13						1	1							1	1			
48	PEN14							1	1							1	1		
49	PEN15								1	1							1	1	
50	R_EIP	-1	-1	-1	-1	-1	-1	-1	-1	-1	-1	-1	-1	-1	-1	-1	-1	-1	-1
51	R_PEI																		
52	R_PEN																		

		Destination															
		18	19	20	21	22	23	24	25	26	27	28	29	30	31	32	33
Source		PEI 0	PEI 1	PEI 2	PEI 3	PEI 4	PEI 5	PEI 6	PEI 7	PEI 8	PEI 9	PEI 10	PEI 11	PEI 12	PEI 13	PEI 14	PEI 15
0	EIP0																
1	EIP1	1															
2	EIP2		1														
3	EIP3			1													
4	EIP4				1												
5	EIP5					1											
6	EIP6						1										
7	EIP7							1									
8	EIP8								1								
9	EIP9									1							
10	EIP10										1						
11	EIP11											1					
12	EIP12												1				
13	EIP13													1			
14	EIP14														1		
15	EIP15															1	
16	EIP16																1
17	EIP17																
18	PEI0																
19	PEI1																
20	PEI2																
21	PEI3																
22	PEI4																
23	PEI5																
24	PEI6																
25	PEI7																
26	PEI8																
27	PEI9																
28	PEI10																
29	PEI11																
30	PEI12																
31	PEI13																
32	PEI14																
33	PEI15																
34	PEN0																
35	PEN1																
36	PEN2																
37	PEN3																
38	PEN4																
39	PEN5																
40	PEN6																
41	PEN7																
42	PEN8																
43	PEN9																
44	PEN10																
45	PEN11																
46	PEN12																
47	PEN13																
48	PEN14																
49	PEN15																
50	R_EIP																
51	R_PEI	-1	-1	-1	-1	-1	-1	-1	-1	-1	-1	-1	-1	-1	-1	-1	-1
52	R_PEN																

		Destination															
		34	35	36	37	38	39	40	41	42	43	44	45	46	47	48	49
Source		PEN 0	PEN 1	PEN 2	PEN 3	PEN 4	PEN 5	PEN 6	PEN 7	PEN 8	PEN 9	PEN 10	PEN 11	PEN 12	PEN 13	PEN 14	PEN 15
0	EIP0	1															
1	EIP1		1														
2	EIP2			1													
3	EIP3				1												
4	EIP4					1											
5	EIP5						1										
6	EIP6							1									
7	EIP7								1								
8	EIP8																
9	EIP9																
10	EIP10									1							
11	EIP11										1						
12	EIP12											1					
13	EIP13												1				
14	EIP14													1			
15	EIP15														1		
16	EIP16															1	
17	EIP17																1
18	PEI0																
19	PEI1																
20	PEI2																
21	PEI3																
22	PEI4																
23	PEI5																
24	PEI6																
25	PEI7																
26	PEI8																
27	PEI9																
28	PEI10																
29	PEI11																
30	PEI12																
31	PEI13																
32	PEI14																
33	PEI15																
34	PEN0																
35	PEN1																
36	PEN2																
37	PEN3																
38	PEN4																
39	PEN5																
40	PEN6																
41	PEN7																
42	PEN8																
43	PEN9																
44	PEN10																
45	PEN11																
46	PEN12																
47	PEN13																
48	PEN14																
49	PEN15																
50	R_EIP																
51	R_PEI																
52	R_PEN	-1	-1	-1	-1	-1	-1	-1	-1	-1	-1	-1	-1	-1	-1	-1	-1

		Destination		
		50	51	52
Source		R_EIP	R_PEI	R_PEN
0	EIP0	1		
1	EIP1	1		
2	EIP2	1		
3	EIP3	1		
4	EIP4	1		
5	EIP5	1		
6	EIP6	1		
7	EIP7	1		
8	EIP8	1		
9	EIP9	1		
10	EIP10	1		
11	EIP11	1		
12	EIP12	1		
13	EIP13	1		
14	EIP14	1		
15	EIP15	1		
16	EIP16	1		
17	EIP17	1		
18	PEI0			
19	PEI1			
20	PEI2			
21	PEI3			
22	PEI4			
23	PEI5			
24	PEI6			
25	PEI7			
26	PEI8			
27	PEI9			
28	PEI10			
29	PEI11			
30	PEI12			
31	PEI13			
32	PEI14			
33	PEI15			
34	PEN0			
35	PEN1			
36	PEN2			
37	PEN3			
38	PEN4			
39	PEN5			
40	PEN6			
41	PEN7			
42	PEN8			
43	PEN9			
44	PEN10			
45	PEN11			
46	PEN12			
47	PEN13			
48	PEN14			
49	PEN15			
50	R_EIP	-1		
51	R_PEI			
52	R_PEN			

1: excitatory (Glutamate: NMDA)

-1: inhibitory ($GABA_A$)

Supplementary Table 3. Synaptic strength between neuron classes

	Destination			
Source	EIP	PEI	PEN	R_{EIP}
EIP		5	6	1
PEI	4			
PEN	6*			
R_{EIP}	5			1.6
R_{PEI}		10		
R_{PEN}			10	

The numbers here indicate the maximum conductance of the synapses formed by the presynaptic neuron class (source) and the postsynaptic neuron class (destination) in each single EB or PB region. If the connection between two neurons covers two EB regions, the maximum conductance of this synaptic connection is twice of the listed value.

*: due to the atypical innervation patterns of EIP0 & EIP17, the synaptic strength between PEN neurons and EIP0/EIP17 neurons are three times of the list value.

Supplementary Table 4a. Strength of input connections* to PB from all LPUs in Drosophila brain

LPU in right hemisphere	Connection strength (A)	LPU in left hemisphere	Connection strength (B)	Total strength A+B
CCP	38.196	ccp	56.152	94.35
DMP	17.037	dmp	28.103	45.14
VMP	12.883	vmp	21.177	34.06
SDFP	0.000	sdfp	23.130	23.13
CVLP	11.068	cvlp	11.378	22.45
MB	2.449	mb	18.000	20.45
AL	11.529	al	6.162	17.69
IDFP	10.459	idfp	1.414	11.87
CMP	5.612	cmp	3.162	8.77
DLP	0.000	dlp	7.313	7.31
SOG	1.000	sog	6.060	7.06
OPTU	0.000	optu	7.049	7.05
PAN	0.000	pan	4.449	4.45
AMMC	1.414	ammc	2.414	3.83
VLP-D	0.000	vlp-d	3.828	3.83
NOD	2.449	nod	0.000	2.45
SPP	0.000	spp	2.000	2.00
LH	0.000	lh	0.000	0.00
LOB	0.000	lob	0.000	0.00
LOP	0.000	lop	0.000	0.00
MED	0.000	med	0.000	0.00
OG	0.000	og	0.000	0.00
VLP-V	0.000	vlp-v	0.000	0.00

*Data are extracted and derived from Shih, C.-T. et al. Connectomics-Based Analysis of Information Flow in the Drosophila Brain. Curr. Biol. 25, 1249–1258 (2015).

Supplementary Table 4b. Top ten strongest input connections* to CCP (right hemisphere) and ccp (left hemisphere)

LPU in right hemisphere	Connection strength (A)	LPU in left hemisphere	Connection strength (B)	Total strength A+B
CCP	38.196	ccp	56.152	94.35
DMP	17.037	dmp	28.103	45.14
VMP	12.883	vmp	21.177	34.06
SDFP	0.000	sdfp	23.130	23.13
CVLP	11.068	cvlp	11.378	22.45
MB	2.449	mb	18.000	20.45
AL	11.529	al	6.162	17.69
IDFP	10.459	idfp	1.414	11.87
CMP	5.612	cmp	3.162	8.77
DLP	0.000	dlp	7.313	7.31
SOG	1.000	sog	6.060	7.06
OPTU	0.000	optu	7.049	7.05
PAN	0.000	pan	4.449	4.45
AMMC	1.414	ammc	2.414	3.83
VLP-D	0.000	vlp-d	3.828	3.83
NOD	2.449	nod	0.000	2.45
SPP	0.000	spp	2.000	2.00
LH	0.000	lh	0.000	0.00
LOB	0.000	lob	0.000	0.00
LOP	0.000	lop	0.000	0.00
MED	0.000	med	0.000	0.00
OG	0.000	og	0.000	0.00
VLP-V	0.000	vlp-v	0.000	0.00

*Data are extracted and derived from Shih, C.-T. et al. Connectomics-Based Analysis of Information Flow in the Drosophila Brain. Curr. Biol. 25, 1249–1258 (2015).

Supplementary Table 4c. Top ten strongest input connections* to VMP (right hemisphere) and vmp (left hemisphere)

LPU in right hemisphere	Connection strength (A)	LPU in left hemisphere	Connection strength (B)	Total strength A+B
CCP	38.196	ccp	56.152	94.35
DMP	17.037	dmp	28.103	45.14
VMP	12.883	vmp	21.177	34.06
SDFP	0.000	sdfp	23.130	23.13
CVLP	11.068	cvlp	11.378	22.45
MB	2.449	mb	18.000	20.45
AL	11.529	al	6.162	17.69
IDFP	10.459	idfp	1.414	11.87
CMP	5.612	cmp	3.162	8.77
DLP	0.000	dlp	7.313	7.31
SOG	1.000	sog	6.060	7.06
OPTU	0.000	optu	7.049	7.05
PAN	0.000	pan	4.449	4.45
AMMC	1.414	ammc	2.414	3.83
VLP-D	0.000	vlp-d	3.828	3.83
NOD	2.449	nod	0.000	2.45
SPP	0.000	spp	2.000	2.00
LH	0.000	lh	0.000	0.00
LOB	0.000	lob	0.000	0.00
LOP	0.000	lop	0.000	0.00
MED	0.000	med	0.000	0.00
OG	0.000	og	0.000	0.00
VLP-V	0.000	vlp-v	0.000	0.00

*Data are extracted and derived from Shih, C.-T. et al. Connectomics-Based Analysis of Information Flow in the Drosophila Brain. Curr. Biol. 25, 1249–1258 (2015).
